# An Attenuated and Highly Immunogenic Variant of the Vaccinia Virus

**DOI:** 10.32607/actanaturae.27384

**Published:** 2024

**Authors:** S. N. Shchelkunov, S. N. Yakubitskiy, K. A. Titova, S. A. Pyankov, I. S. Shulgina, E. V. Starostina, M. B. Borgoyakova, D. N. Kisakov, L. I. Karpenko, G. A. Shchelkunova, A. A. Sergeev

**Affiliations:** State Research Center of Virology and Biotechnology “Vector”, Rospotrebnadzor, Koltsovo, Novosibirsk region, 630559 Russian Federation

**Keywords:** vaccinia virus, orthopoxviruses, targeted gene deletion, vaccination, intradermal injection, immunogenicity, protectivity

## Abstract

The vaccinia virus (VACV) has been used for prophylactic immunization against smallpox for many decades. However, the VACV-based vaccine had been highly reactogenic. Therefore, after the eradication of smallpox, the World Health Organization in 1980 recommended that vaccination against this infection be discontinued. As a result, there has been a rise in the occurrence of orthopoxvirus infections in humans in recent years, with the most severe being the 2022 monkeypox epidemic that reached all continents. Thus, it is crucial to address the pressing matter of developing safe and highly immunogenic vaccines for new generations to combat orthopoxvirus infections. In a previous study, we created a LAD strain by modifying the LIVP (L) VACV strain, which is used as a first-generation smallpox vaccine in Russia. This modification involved introducing mutations in the *A34R *gene to enhance extracellular virion production and deleting the *A35R *gene to counteract the antibody response to the viral infection. In this study, a strain LADA was created with an additional deletion in the DNA of the LAD strain *ati *gene. This *ati *gene directs the production of a major non-virion immunogen. The findings indicate that the LADA VACV variant exhibits lower levels of reactogenicity in BALB/c mice during intranasal infection, as compared to the original L strain. Following intradermal immunization with a 105 PFU dose, both the LAD and LADA strains were found to induce a significantly enhanced cellular immune response in mice when compared to the L strain. At the same time, the highest level of virus-specific IFN-γ producing cells for the LAD variant was detected on the 7^th^ day post-immunization (dpi), whereas for LADA, it was observed on 14 dpi. The LAD and LADA strains induced significantly elevated levels of VACV-specific IgG compared to the original L strain, particularly between 28 and 56 dpi. The vaccinated mice were intranasally infected with the cowpox virus at a dose of 460 LD_50_ to assess the protective immunity at 62 dpi. The LADA virus conferred complete protection to mice, with the LAD strain providing 70% protection and the parent strain L offering protection to only 60% of the animals.

## INTRODUCTION

The dangerous for humans smallpox virus (*Variola virus*, VARV) and its related zoonotic counterparts, the monkeypox (*Monkeypox virus*, MPXV), cowpox (*Cowpox virus*, CPXV), camelpox (*Camelpox virus*, CMLV), and vaccinia (*Vaccinia virus*, VACV) viruses, are all part of the *Orthopoxvirus *genus within the Poxviridae family [1].

Immunizing humans or animals with a low-virulent replicating variant or a weakly pathogenic virus is the most effective method of preventing viral diseases. The earliest recorded form of protection against infectious diseases involved smallpox vaccination [2].

VACV-based vaccines lack significant species specificity towards orthopoxviruses, by which they enable immunization against any type of orthopoxvirus, thus preventing infectious disease outbreaks in both humans and animals [3].

The first-generation smallpox vaccine consisted of VACV, which was propagated by replicating the virus in the epidermis of calves or other animals. In today’s conditions VACV vaccine strains are manufactured using mammalian cell cultures, and they are known as second-generation smallpox vaccines [4, 5].

The World Health Organization strongly recommended discontinuing vaccination after the declaration of the eradication of smallpox around the world in 1980. The decision to discontinue was due to the severe adverse reactions, including fatalities, that were associated with the first-generation live vaccine [1].

The discontinuation of smallpox vaccination has resulted in a notable absence of immunity against zoonotic orthopoxvirus infections among a substantial proportion of individuals, predominantly those under the age of 40–45. Given the rising number of human infections caused by orthopoxviruses, particularly the monkeypox virus, it is crucial to reconsider the potential re-emergence of smallpox or a similar illness through the natural evolution of these viruses [6, 7].

In order to minimize the risk of emergence of highly pathogenic human orthopoxvirus resulting from natural evolution and prevent localized outbreaks from spreading into global epidemics, researchers should concentrate efforts on creating safe new generations of live vaccines based on VACV [3, 8].

The production of third-generation attenuated smallpox vaccines involves the serial passages of a specific VACV strain in a cell culture of a heterologous host. For instance, the well-documented thirdgeneration MVA smallpox vaccine is obtained by subjecting the Ankara VACV strain to a significant number of passages on chicken fibroblast cultures. The genome of the MVA strain underwent multiple mutations and extensive deletions in relation to the DNA of the original VACV strain. MVA is distinguished by its failure to replicate in the majority of mammalian cells, including human cells [9].

A novel strategy for obtaining attenuated replicating fourth-generation smallpox vaccines involves the introduction of targeted mutations or deletions/insertions into the genes that regulate the body’s antiviral defense mechanisms via genetic engineering techniques.

Extensive research on gene deletion of immunomodulatory factors in VACV has enabled the identification of specific genes that, upon inactivation, have led to virus attenuation. Various attempts have been made to generate attenuated and highly immunogenic VACV strains through the targeted inactivation of one or several viral genes. However, clinical application of these thus-obtained VACV variants has not followed [3, 4, 10].

We created a recombinant variant of VACΔ5 by modifying the LIVP (L) VACV strain, the first-generation smallpox vaccine used in Russia. This modification involved disrupting five virulence genes, namely hemagglutinin (*A56R*), gamma interferonbinding protein (*B8R*), thymidine kinase (*J2R*), complement- binding protein (*C3L*), and Bcl-2-like inhibitor of apoptosis (*N1L*). It has been demonstrated that deactivating specific virulence genes does not impact the ability of VACV to reproduce in mammalian cell cultures. Characterization of the obtained strain VACΔ5 revealed a notable decrease in reactogenicity and neurovirulence compared to the original L strain [11]. To increase the production of virus-specific antibodies, the *A35R *gene additionally was inactivated in the VACΔ5 genome. The protein product of this gene impedes the presentation of antigens by major histocompatibility complex class II, the activation of T-lymphocytes, and the subsequent generation of chemokines and cytokines. Upon introduction into mice, the created variant VACΔ6 triggered a notably heightened production of virus-neutralizing antibodies and afforded more quality protection than the original L strain [12]. Following preclinical studies [13] and clinical trials, the OrthopoxVac vaccine (VACΔ6) was officially licensed in Russia in November 2022 [2], achieving a significant milestone as the world’s first fourth-generation vaccine targeting human orthopoxvirus infections.

Given that VACV encompasses an extensive range of genes responsible for viral progeny formation and immune response regulation to viral infection [10, 14], our research has persisted in developing novel attenuated and highly immunogenic VACV variants through genetic engineering techniques.

The aim of this study was to generate a recombinant LADA variant derived from the L VACV strain. This variant contained specific mutations in the *A34R *gene to enhance the production of extracellular virions. Additionally, the *A35R *gene, which inhibits the antibody response to viral infection, was deleted and the *ati *gene, directing the production of a major non-virion immunogen, was deleted too. Furthermore, we conducted an examination in a mouse model to evaluate the reactogenicity and kinetics of the immune response development specific to VACV vaccination.

## EXPERIMENTAL SECTION

**Viruses, cell culture**

In this study, we utilized Clone 14 of the LIVP strain VACV (L) [11], the LIVP-A34R*-dA35R (LAD) strain derived from it [15], and the GRI-90 CPXV strain [16]. The viruses were cultured and titrated on the CV-1 African green monkey kidney cell line from the SRC VB Vector cell culture collection.

Generation of the recombinant LIVP-A34R*-dA35R-ati strain

For the targeted deletion of the *ati *VACV gene, a monolayer of CV-1 cells was infected with the LAD strain and subsequently transfected with the recombinant plasmid pΔati under the gpt-selection conditions specified for VACV recombinants in the earlier study [17]. PCR analysis and subsequent sequencing of viral DNA allowed us to identify the target virus variant LIVP-A34R*-dA35R-ati (LADA).

**The animals**

The BALB/c mice used in this research were obtained from the breeding animal facility of the SRC VB Vector. All the experimental animals were housed under veterinary legislation, receiving a standard diet and access to adequate water. We adhered to the ethical principles governing the use of animals in experimental studies. The animal manipulations were conducted with the consent of the Bioethics Committee of the SRC VB Vector (Protocol No. 02-06.2022).

**Immunization of mice and subsequent sampling for assays**

BALB/c mice, aged 6–7 weeks, were immunized with VACV strains (L, LAD, or LADA) (28 animals per group, virus dose 105 plaques forming units (PFU)/20 µL/mouse) via intradermal injection into the dorsal side of the tail, approximately 1 cm from the base [18]. In order to establish a negative control, mice received an injection of saline solution.

The humoral and cellular immune responses in mice were analyzed at 7, 14, and 21 days post-immunization (dpi). Six mice from each group were selected for inclusion in the analysis for every specified time point. Blood was extracted from the retroorbital venous sinus in mice using a 23G × 1.25 needle. The serum was obtained by subjecting individual animal blood samples to centrifugation at a relative centrifugal force of 1000 g for 10 minutes, thereby precipitating the blood cells. The resulting sera were subjected to incubation at a temperature of 56°C for 30 minutes and subsequently stored at a temperature of –0°C.

Following blood collection at 7, 14, and 21 dpi, mice were euthanized by cervical dislocation. Individual spleens were aseptically extracted from each of the six mice in the respective study groups at the corresponding time point.

Blood samples were collected from the retroorbital venous sinus of the same mice (ten mice from each group) at 28, 42, and 56 dpi.

**Splenocyte isolation**

Splenocytes were isolated by wiping an individual spleen through 70 and 40 μm cell filters (BD Falcon™ USA) using a syringe piston. Once the erythrocytes were removed using an erythrocyte lysis buffer (Sigma, USA), the splenocytes were washed and then resuspended in an RPMI-1640 nutrient medium. The medium was enriched with 2 mM *L*-glutamine and gentamicin at a concentration of 50 μg/mL. The determination of cell viability and concentration was conducted using a trypan blue dye (Bio-Rad, USA) on an automatic cell counter TC20 (Bio-Rad).

**Quantification of IFN-γ-producing cells**

The T-cell immune response intensity in the immunized mice was assessed by quantifying the number of IFN-γ-producing splenocytes through the IFN-γ ELISpot technique. The experiment was conducted utilizing the Murine IFNγ ELISPOT Kit (with precoated plates) obtained from Abcam, USA, following the guidelines provided by the manufacturer. The splenocytes were cultured in the Lymphogen medium (“PanEco”, Russia) with a cell density of 105 cells per well. The cells were stimulated using a combination of VACV-specific immunodominant peptides, namely SPYAAGYDL, SPGAAGYDL, VGPSNSPTF, KYGRLFNEI, GFIRSLQTI, KYMWCYSQV, and SFIRSLQNI, each at a concentration of 20 μg/mL [19, 20]. The mitogenic activity was induced using Concanavalin A, with the Lymphogen medium as the negative control. The IFN-γ-producing cells were quantified using an ELISpot reader (Carl Zeiss, Germany).

**Enzyme immunoassay of mouse blood sera**

The performance of the enzyme-linked immunosorbent assay (ELISA) on individual mouse sera followed the guidelines provided in [18]. The antigen employed in this study was derived from the purification of the virions of strain L VACV using centrifugation with a sucrose cushion. The mouse serum samples underwent titration through a series of twofold serial dilutions, ranging from 1 : 100 to 1 : 12800. The ELISA titration was repeated on the following day. The determination of IgG titers was accomplished using mouse anti-IgG peroxidase conjugates obtained from Sigma, USA. The IgG titers of each serum sample were determined for each repetition individually, and then a mean value was calculated. The geometric mean values of the logarithms of the VACV-specific IgG reverse titer were computed for each experimental group. Additionally, confidence intervals were determined at the 95% probability level to assess the likelihood of each sample matching the general population.

**Assessment of the degree of protective immunity in immunized mice**

On dpi 62, the groups that had received immunization with the L, LAD, or LADA strains, as well as the control animals, were infected intranasally (i.n.) with CPXV GRI-90. The infection was administered at a dose of 460 LD_50_ (2.0 × 10^6^ PFU/50 μL/mouse), with 10 animals in each group. A 14-day monitoring period was observed to record the clinical manifestations of infection and mortality in the animals.

To evaluate the presence of disease symptoms, we utilized a scoring scale that encompassed the following values: 0 – no signs of disease; 1 – slight hair ruffling; 2 – severe hair ruffling; 3 – severe hair ruffling, as well as slouching posture or conjunctivitis; 4 – difficulty in breathing or lack of movement; and 5 – death.

We conducted individual weighing sessions of the mice every two days. The arithmetic mean body weights for each group of mice at each time point were determined and then expressed as a percentage of the initial weight.

The data were collected from groups of animals that received immunization with the VACV variants under investigation, as well as from the groups of mice that were not immunized and remained uninfected (Negative Control, N.C.), or were infected with CPXV GRI-90 (Positive Control, P.C.).

**Assessment of the pathogenicity of VACV strains**

In order to study the pathogenicity of the L and LADA VACV strains through i.n. infection, we utilized 3-week-old BALB/c mice weighing 10–12 g. Each group contained 10 animals. Following inhalation anesthesia with diethyl ether, the mice received an injection of virus-containing liquid (50 μL, dose 107 PFU/mouse) or a saline solution (control group) into their nasal cavity. The animals were under observation for 14 days, during which their deaths were documented.

**Statistical data analysis**

The statistical processing and comparison of the results were conducted using the standard methods provided by the Statistica 13.0 computer program package (StatSoft Inc. 1984–2001). A *P *value below 0.05 was deemed to be statistically significant.

## RESULTS

**Cellular immune response to vaccination of mice with VACV variants**

**Fig. 1:**
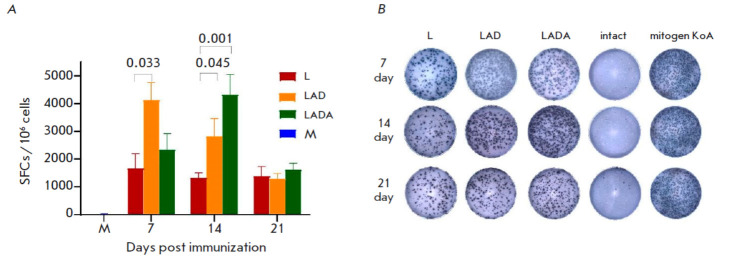
The results of the ELISpot analysis of the VACV-specific cellular response in BALB/c mice immunized with the L, LAD, or LADA viruses. M – the control mice (not immunized). (*A*) – the number of splenocytes expressing IFN-γ in response to stimulation with a pool of VACV-specific peptides, per million splenocytes. The data are presented as medians along with their respective ranges. Graphical and statistical analysis was performed using the GraphPad Prism 9.0 software. The *P *values are indicated above the brackets. (*B*) – the representative images of ELISpot wells

The vaccination was performed on adult BALB/c mice, aged 6–7 weeks, through intradermal injection. The mice were given the L, LAD, or LADA VACV strains at a dose of 105 PFU per animal. Following the time points 7, 14, and 21 dpi, the mice (six animals per group after vestibular blood collection) were euthanized. Spleens were then extracted, and splenocytes were isolated. The quantification of IFN-γ- producing cells in each animal, following stimulation with a pool of VACV-specific peptides, was conducted using ELISpot. As outlined in *Fig. 1*, the results indicate significant discrepancies in the cellular immune response development dynamics and levels among the three VACV variants investigated in the laboratory mice after intradermal immunization.

**Humoral immune response to vaccination of mice with VACV variants**

Blood samples were collected from BALB/c mice aged 6–7 weeks, which had been intradermally immunized with the L, LAD, or LADA VACV strains at a dose of 105 PFU. The samples were taken from the retroorbital venous sinus at 7, 14, 21, 28, 42, and 56 dpi, and sera were obtained at the same time points. The blood samples were collected from six animals in each group at three designated time points, specifically 7, 14, and 21 dpi. The blood samples were collected from the same animals (10 mice per group) at 28, 42, and 56 dpi. The ELISA method was used to determine the VACV-specific IgG titers in each serum sample.

**Fig. 2:**
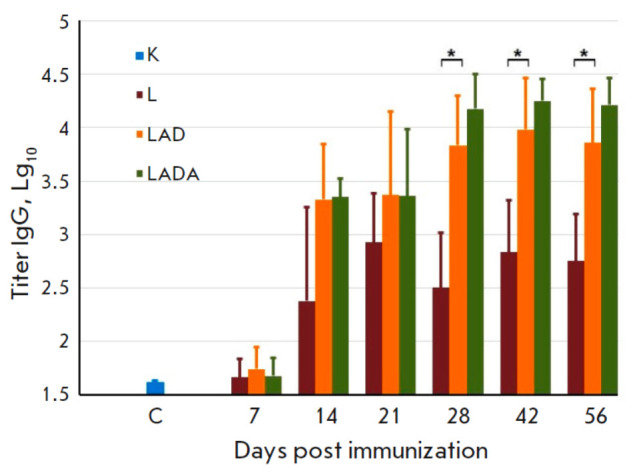
The titers of the VACV-specific IgG in the sera of mice immunized with the L, LAD, or LADA viruses. C – the blood serum of mice injected with saline. The data are presented as medians along with their respective ranges. Graphical and statistical analysis was performed using the GraphPad Prism 9.0 software. *Statistically significant differences with *P * < 0.05

The results depicted in *Fig. 2* provide evidence that the recombinant strains LAD and LADA effectively stimulate the production of VACV-specific IgG, surpassing the levels achieved by the parental strain L, starting from 14 dpi. Notably, the LADA strain was found to produce the highest level of antibodies between 28–56 dpi.

**Protective efficacy in immunized mice against a lethal orthopoxvirus infection**

**Fig. 3:**
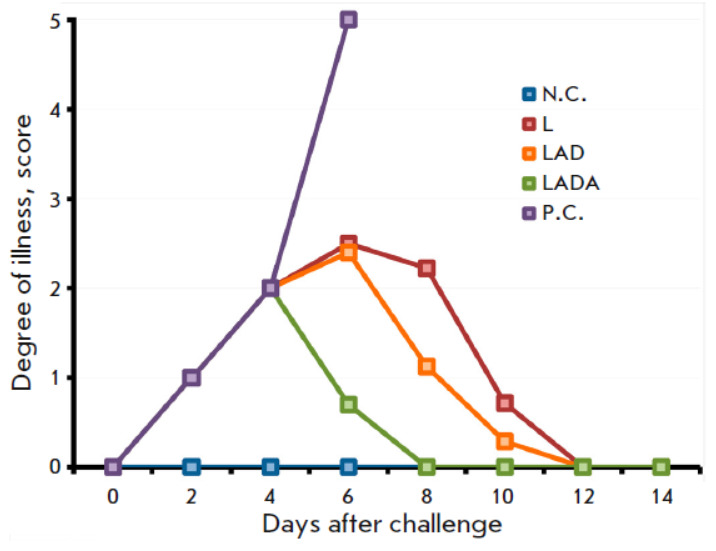
The dynamics of the clinical manifestations of the infection in mice vaccinated with the L, LAD, or LADA viruses at a dose of 105 PFU after intranasal infection with CPXV GRI-90 at a dose of 460 LD_50_ on day 62 after immunization. The data are presented for groups comprising 10 animals that were immunized with the respective viruses, as well as groups that were neither immunized nor infected (N.C.) or infected with CPXV (P.C.)

**Fig. 4:**
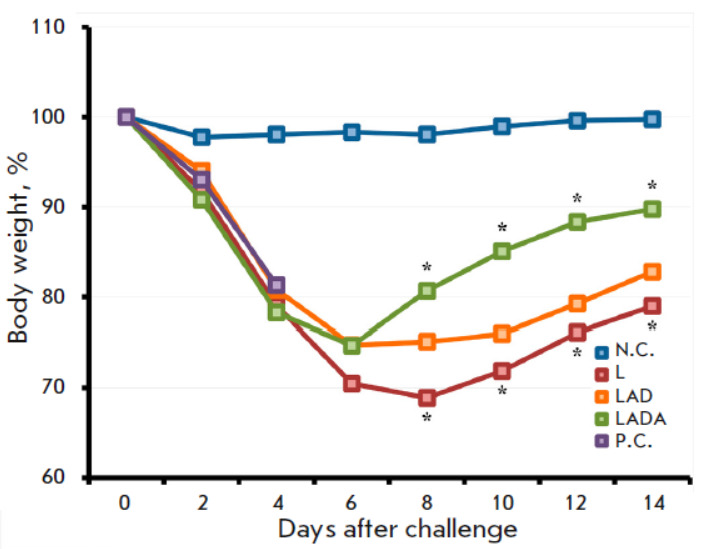
The dynamics of the changes in body weight in mice vaccinated with the L, LAD, or LADA viruses at a dose of 105 PFU after their intranasal infection with CPXV GRI-90 at a dose of 460 LD_50_ on day 62 after immunization. The data are presented for groups comprising 10 animals that were immunized with the respective viruses, as well as groups that were neither immunized nor infected (N.C.) or infected with CPXV (P.C.). *Statistically significant differences with *P * < 0.05 in the mean values between the LADA and L groups

**Fig. 5:**
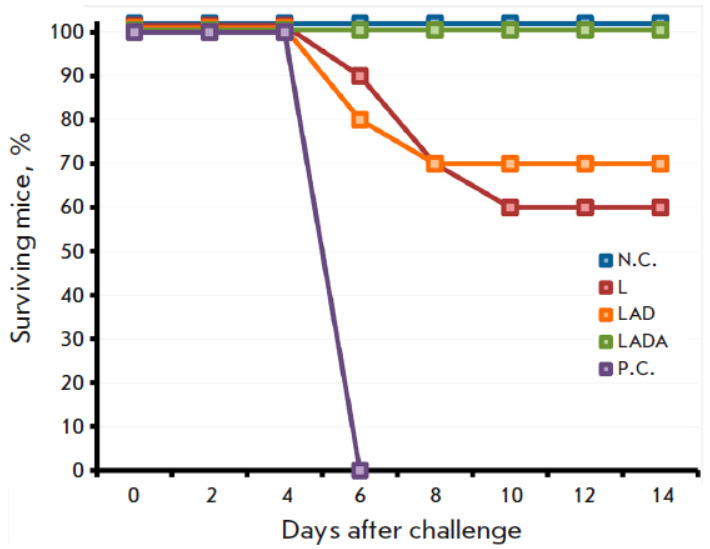
The dynamics of the death of mice vaccinated with the L, LAD, or LADA viruses at a dose of 105 PFU after their intranasal infection with CPXV GRI-90 at a dose of 460 LD_50_ on day 62 after immunization. The data are presented for groups comprising 10 animals that were immunized with the respective viruses, as well as groups that were neither immunized nor infected (N.C.) or infected with CPXV (P.C.)

This study aimed to assess the impact of the L, LAD, and LADA strains on the development of protective immunity against a lethal infection of mice with heterologous orthopoxvirus. For this purpose, the cohorts of immunized and control (non-immunized) animals were exposed to CPXV GRI-90 at a dosage of 460 LD_50_ on 62 dpi. All experimental groups exhibited signs of viral infection (*Fig. 3*), along with the corresponding change to the body weight of the animals (*Fig. 4*). Vaccination with the LADA strain produced the least pathogenic effect of CPXV on the mice (*Fig. 3* and *Fig. 4*). The survival rate of all the animals in this particular group was 100%, whereas in the groups of mice vaccinated with the LAD or L strains, the survival rates were 70% and 60%, respectively (*Fig. 5*).

**Pathogenic properties of the L and LADA strains in an intranasal infection of mice**

**Fig. 6:**
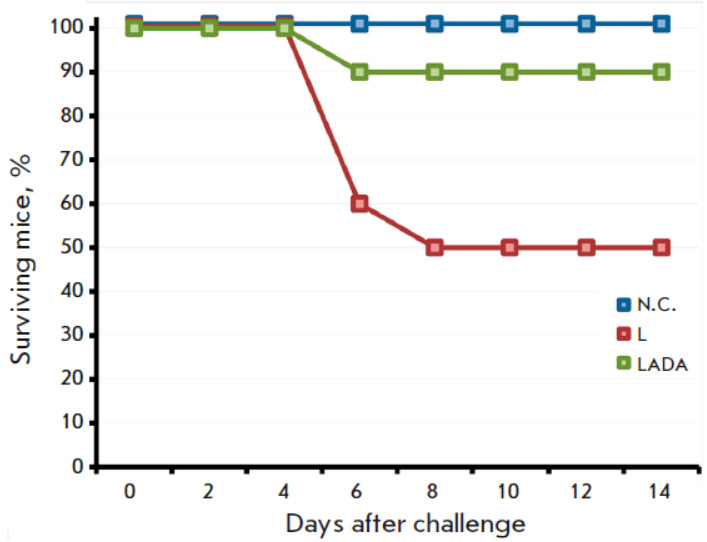
The dynamics of the death of mice after intranasal infection with the L or LADA viruses at a dose of 107 PFU. N.C. – the mice that were intranasally injected with saline. The data are presented for groups comprising 10 animals

The pathogenicity of the L and LADA strains was investigated in this study using 3-week-old BALB/c mice, with 10 animals in each group. The mice were intranasally infected with viruses at a dosage of 107 PFU per animal. The animals were closely monitored over a two-week period, and any instances of mortality were documented. The mortality rate was significantly higher in the group of mice infected with VACV strain L, with 50% of the animals dying, compared to the group infected with strain LADA, where only 10% of the animals died (*Fig. 6*).

## DISCUSSION

In the process of coevolution with vulnerable animals, orthopoxviruses have developed diverse molecular mechanisms to suppress specific stages of innate and adaptive immune responses to infection [10]. The genes that govern the immune response are generally nonessential and have no bearing on the ability of viruses to multiply in cell cultures. Consequently, the targeted inactivation or modification of these genes may prove to be a fruitful approach to acquiring attenuated and highly immunogenic variations of VACV [10, 14, 21, 22, 23, 24, 25].

In our prior studies, we examined the progression of humoral and T-cell immune reactions in mice that were vaccinated with VACV variants containing a mutant *A34R *gene, resulting in heightened extracellular virion production or a deleted *A35R *gene, leading to the inhibition of antigen presentation by major histocompatibility complex class II, inducing immune priming of T-lymphocytes and subsequent synthesis of chemokines and cytokines. The simultaneous modification of the *A34R *gene and deletion of the *A35R* gene produced a synergistic impact on the immunogenic properties of the LAD strain of VACV surpassing those of the parental strain L [15].

Additionally, we investigated the influence of the* ati *gene-encoded non-virion major immunogenic protein production on the manifestation of VACV pathogenicity and immunogenicity [17]. The targeted removal of the *ati *gene resulted in heightened production of VACV-specific IgG in the obtained virus variant LIVPΔati, following the immunization of mice. This increase in IgG production was significantly greater than what was observed with vaccination using the parental L strain. Moreover, immunization with LIVPΔati provided enhanced protection against a subsequent orthopoxvirus infection.

Within this investigation, a modified variant LADA was generated by introducing mutations into the* A34R *gene to enhance extracellular virion production, eliminating the *A35R *gene to suppress its inhibition of the antibody response to viral infection, and deleting the *ati *gene responsible for the synthesis of major non-virion immunogen that does not possess viral neutralizing properties. The investigation into the properties of the LADA strain using an intranasally infected mouse model demonstrated that the resulting VACV variant displays attenuation when compared to the original L strain (*Fig. 6*).

The LAD and LADA strains elicited a more pronounced cellular immune response in mice when they were immunized intradermally with a dose of 105 PFU, as compared to the L strain (*Fig. 1*). The highest number of cells producing virus-specific IFN-γ was observed at 7 dpi for the LAD variant, while for LADA it was detected at 14 dpi. The change in the number of IFN-γ-producing cells observed in LADA seems to be caused by the absence of synthesis of a major non-virion immunogen.

Starting at 14 dpi, production of VACV-specific IgG was observed for all strains (*Fig. 2*). Significantly increased levels of specific antibodies were observed in response to the recombinant LAD and LADA variants as compared to the parental L strain, particularly within the 28–56 dpi period. It should be noted that the LADA strain demonstrated the highest levels of VACV-specific antibodies from 28–56 dpi.

The protective immunity conferred by vaccination with the VACV variants was evaluated by infecting the mice with a highly lethal dose of heterologous CPXV at 62 dpi (460 LD_50_). The LADA virus provided comprehensive protection (*Fig. 5*), resulting in minimal clinical manifestations of infection on the 2^md^ to 6th day (*Fig. 3*) and a significantly lesser temporary decrease in body weight compared to the other experimental groups of mice (*Fig. 4*). In the same conditions, the parental strain L offered 60% protection whereas the LAD strain showed a higher protection rate of 70%.

Thus, the created LADA variant is attenuated and more immunogenic compared to the L strain, on the basis of which a first-generation smallpox vaccine had been obtained and approved for clinical use in Russia.

Based on these findings, the *A35R *and *ati *genes can be regarded as potential candidates for the integration of target genes into the DNA of the LIVP-A34R* strain, thereby generating safe and efficacious live polyvalent VACV-derived vaccines.

## Abbreviations

CPXV: cowpox virus
VACV: vaccinia virus
PFU: plaque forming units
dpi: day post-immunization
i.d.: intradermal
i.n.: intranasal
